# Evolving small spiking neural networks to work as state machines for temporal pattern recognition

**DOI:** 10.1186/1471-2202-16-S1-P238

**Published:** 2015-12-18

**Authors:** Borys Wróbel, Ahmed Abdelmotaleb, Neil Davey, Volker Steuber

**Affiliations:** 1 Evolutionary Systems Group, Adam Mickiewicz University, Poznan, Poland; 2 Systems Modeling Group, IOPAN, Sopot, Poland; 3 Biocomputation Research Group, University of Hertfordshire, Hatfield, UK

## 

The mechanisms that allow biological networks to recognize temporal patterns of spikes that encode sensory inputs are unclear. Here we extend our previous work [1], using an artificial life software platform, GReaNs [2] to evolve spiking neural networks as state machines to recognize temporal patterns of spikes.

GReaNs implements a genetic algorithm to obtain the topology of the networks (and the weights of the synaptic connections), starting from a population of networks of neurons connected randomly. The encoding of the neural networks in the genome is inspired by the encoding of genetic networks in biological genomes; neurons in GReaNs are modeled as either leaky integrate and fire neurons with a fixed threshold (LIF) or adaptive-exponential integrate and fire neurons [2]. The number of neurons in the network is not limited in GReaNs, but here as previously [1] we limit the size of the networks so that the analysis of the way the networks function is simplified.

In the computational task we consider, the network has several input neurons and one output neuron. A spike or burst received by an input neuron corresponds to a certain symbol (for example, sound with a specific frequency; a flash of light with a specific color). The output neuron should be active only after the network receives a certain sequence of symbols (a temporal pattern).

Our preliminary results with LIF networks with a fixed threshold networks suggest that the presence of recurrent connections in the network allows the interneurons to reach plateau subthreshold states that provide a memory of what symbols have been received thus far. Here we will investigate the robustness of this solution to noise in the network, and then discuss the possibility to extend the paradigm to evolve spiking networks to accept regular languages.
